# Characteristics of Taiwanese patients of PNH in the international PNH registry

**DOI:** 10.1186/s12959-016-0094-0

**Published:** 2016-10-04

**Authors:** Wen-Chien Chou, Wei-Han Huang, Ming-Chung Wang, Chao-Sung Chang, Shih-Peng Yeh, Tzeon-Jye Chiou, Yeu-Chin Chen, Tseng-Hsi Lin, Ming-Ching Shen, on behalf of the Taiwan PNH study group

**Affiliations:** 1 Department of Laboratory Medicine, National Taiwan University Hospital, Taipei, Taiwan; 2 Department of Hematology and Oncology, Hualien Tzu Chi Hospital, Hualien, Taiwan; 3 Division of Hematology and Oncology Department of Medicine, Kaohsiung Chang Gung Memorial Hospital, Kaohsiung, Taiwan; 4 Department of Hematology and Oncology, Kaohsiung Medical University Hospital, Kaohsiung, Taiwan; 5 Department of Hematology and Oncology, China Medical University Hospital, Taichung, Taiwan; 6 Department of Medicine and Division of Transfusion Medicine Taipei, Veterans General Hospital, Taipei, Taiwan; 7 Division of Hematology and Oncology, Department of Medicine, Tri-Service General Hospital, Taipei, Taiwan; 8 Department of Hematology and Oncology, Taichung Veterans General Hospital, Taichung, Taiwan; 9 Department of Hematology and Oncology, Changhua Christian Hospital, Changhua, Taiwan

**Keywords:** Paroxysmal nocturnal hemoglobinuria, Taiwan

## Abstract

**Background:**

Paroxysmal nocturnal hemoglobinuria (PNH) is a rare and acquired hematopoietic stem cell disease, with florid clinical presentations. Although this disease has been characterized in the western countries, its clinical and laboratory features in Taiwan have not yet been reported.

**Results:**

As a part of an international prospective, non-interventional, observational registration trial of PNH, we have analyzed 63 patients recruited between 2009 and 2015 in Taiwan, with comparison to the 3857 patients in the rest of the world (ROW). The median age of diagnosis of our patients is 46 (range 9–84), without sex preponderance. While most of the clinical and laboratory presentations of our patients are similar to the ROW, ours have higher lactate dehydrogenase levels, lower hemoglobin, and higher frequencies of symptoms including shortness of breath and erectile dysfunction at the time of diagnosis. The incidence of thromboembolism was not statistically different between ours and the ROW (6.7 % vs 13.5 %, *P* = 0.178). The patients in Taiwan were treated more frequently with corticosteroid (53.2 % vs 32 %, *P* < 0.001), but less frequently with cyclosporine/anti-thymocyte globulin and heparin/warfarin, both *P* < 0.001).

**Conclusions:**

This is the first systematic review on the Taiwanese PNH patients. Our analysis would provide key information about our PNH patients and would help understanding the basic characteristics of this rare disease in Taiwan.

**Trial registration:**

This trial has been registered to ClinicalTrails.gov NCT01374360.

## Background

Paroxysmal nocturnal hemoglobinuria (PNH) is a rare, acquired chronic disease characterized by complement-mediated hemolysis. PNH patients may suffer from life threatening complications such as thromboembolism (TE), pulmonary hypertension and impaired renal function; according to a western study, up to 35 % of PNH patients die within 5 years of diagnosis [1]. However, the clinical features of PNH patients in Taiwan have not yet been characterized.

The International PNH Registry is a worldwide, observational, non-interventional study collecting safety and efficacy of eculizumab, as well as other clinical data from PNH patients, irrespective of treatment. This registry aims to enhance the understanding of the natural history of this rare disease, hoping to improve the diagnosis and optimize patient management and outcomes.

The natural history of PNH has been investigated via some analyses with different population sizes [1–5]. Schrezenmeier et al. reported the characteristics of 1610 patients in a global registry [6]. It is the first time now that we look at the baseline characteristics of this disease in Taiwanese PNH patients in this global PNH registry. The data include demographics, clinical characteristics as well as concomitant medications. We describe the similarities and differences between Taiwanese PNH population and the rest of the world (ROW).

## Methods

### Patient population

The international PNH registry is a prospective, non-interventional, observational study. The Registry collects information from patients monitored in current medical practice irrespective of past, present, or future treatment policies. The registry was approved by the institutional review boards (or equivalent) of participating centers, and all patients provided written informed consent prior to inclusion. The registry is sponsored by Alexion Pharmaceuticals, Inc., and is overseen by an independent executive committee of international PNH experts.

Patients of any age with a clinical diagnosis of PNH confirmed by Ham test or Sucrose-Lysis test in the pre-flow cytometry era and/or detectable PNH clone of 0.1 % or more were eligible for this registry in Taiwan. A total of 63 PNH patients were enrolled in 11 institutes in Taiwan from 2009 to 2015.

### Data collection

Data is collected via the case report form (CRF). Data captured in the registry include patients’ demographics, medical history, comorbidities, disease characteristics, symptoms, transfusion requirements, thrombotic events, and treatments. The lab data captured include lactate dehydrogenase (LDH) levels, hemoglobin levels, PNH clone sizes, renal function, and others. PNH-related symptoms, including abdominal pain, dyspnea, short of breath, erectile dysfunction, fatigue and hemoglobinuria were collected via history taking by the physicians.

Baseline is defined as the start date of eculizumab for treated patients or registry enrollment date for other patients. Analyses are performed with available data for each parameter. The number of patients contributing data for each assessment might not be consistent. For parameters that are collected only after enrollment, the available population will reflect the patients who started treatment after enrollment. Continuous variables were described using standard summary statistics; categorical variables were described using frequencies and percentages. *P* < 0.05 was considered statistically significant.

## Results

### Patients’ demographics and clinical characteristics

As of 2 Dec 2015, 63 patients from 11 centers in Taiwan were enrolled in the international PNH registry. 100 % of patients were from Asia. Patients’ demographics and clinical characteristics of the 63 enrolled patients are provided in Table 1.

**Table 1:** Patients’ demographics and clinical characteristics at enrollment into the International PNH Registry, Taiwan versus the rest of the world (ROW)

Parameter	Taiwan Patients (*n* = 63)	ROW (*n* = 3857)	*p*-value
Age, years, median (range)	46.0 (9, 84)	43.0 (0, 104)	0.331
Females, *n* (%)	31 (49.2)	2072 (53.7)	0.525
Age at disease start, years, median (range)	37.5 (8, 84) (*n* = 62)	34.0 (0, 90) (*n* = 3773)	0.483
Disease duration, years, median (range)	4.5 (0.1, 34.8) (*n* = 62)	3.0 (0.0, 100.4) (*n* = 3773)	0.890
GPI-Deficient Granulocytes, *n* (%)	(*n* = 11)	(*n* = 1619)	0.980
< 10 %	3 (27.3)	482 (29.8)	
> =10 % to < 50 %	1 (9.1)	317 (19.6)	
> = 50 %	7 (63.6)	820 (50.6)	
LDH Ratio (xULN), median (Q1, Q3)	4.9 (1.9, 9.1) (*n* = 25)	2.0 (1.0, 5.2) (*n* = 2398)	0.005*
> = 1.5, *n* (%)	20 (80.0)	1406 (58.6)	0.039*
Hemoglobin (g/L), median (Q1, Q3)	80.0 (68.0, 105.0) (*n* = 51)	99.0 (84.0, 118.0) (*n* = 2996)	<0.001*
Platelets (x10^9^/L), median (Q1, Q3)	112.0 (51.0, 186.0) (*n* = 51)	114.0 (51.0, 176.0) (*n* = 2990)	0.680
Serum Creatinine (umol/L), median (Q1, Q3)	66.3 (53.0, 97.2) (*n* = 36)	73.0 (61.9, 91.0) (*n* = 2746)	0.36
Proteinuria, *n* (%)			0.056
Trace	2 (12.5)	93 (10.0)	
1+ to 4+	6 (37.5)	158 (16.9)	
eGFR (mL/min), median (Q1, Q3)	95.8 (70.1, 116.5) (*n* = 36)	95.6 (73.9, 114.1) (*n* = 2638)	0.994
< 30, *n* (%)	3 (8.3)	62 (2.4)	0.143
30– < 60	3 (8.3)	329 (12.5)	
60– < 90	8 (22.2)	726 (27.5)	
> =90	22 (61.1)	1521 (57.7)	
Neutrophils (x 10^9^/L), median (Q1, Q3)	2.0 (1.0, 2.9) (*n* = 27)	1.9 (1.1, 2.9) (*n* = 2674)	0.696
History of Abdominal Pain, *n* (%)	17 (31.5) (*n* = 54)	943 (33.5) (*n* = 2817)	0.884
History of Dysphagia, *n* (%)	7 (13.0) (*n* = 54)	434 (15.4) (*n* = 2812)	0.848
History of Shortness of Breath, *n* (%)	32 (59.3) (*n* = 54)	1180 (41.9) (*n* = 2819)	0.012*
History of Erectile Dysfunction, *n* (%)	17 (60.7) (*n* = 28)	271 (23.0) (*n* = 1180)	<0.001*
History of Fatigue, *n* (%)	44 (81.5) (*n* = 54)	2214 (78.5) (*n* = 2820)	0.738
History of Hemoglobinuria, *n* (%)	37 (68.5) (*n* = 54)	1288 (45.7) (*n* = 2816)	0.001*
History of Bone Marrow Disorder, *n* (%)	36 (58.1) (*n* = 62)	2265 (60.2) (*n* = 3762)	0.794
History of TE, *n* (%)	4 (6.7) (*n* = 60)	502 (13.5) (*n* = 3710)	0.178
History of any RBC Transfusion, *n* (%)	40 (67.8) (*n* = 59)	1816 (57.0) (*n* = 3185)	0.274
History of Impaired Renal Function, *n* (%)	7 (12.1) (*n* = 58)	456 (12.6) (*n* = 3609)	>0.999
History of Pulmonary Hypertension, *n* (%)	0 (0.0) (*n* = 58)	68 (1.9) (*n* = 3670)	0.627
Ever Treated with Eculizumab, *n* (%)	25 (39.7)	1666 (43.2)	0.610

*p <0.05

The median patient age at enrollment was 46 years (rang 9–84 years), and 49.2 % of patients were female. Median disease duration from disease onset was 4.5 years (range from 0.1 to 34.8 years).

Similar to the ROW, most of the PNH patients in Taiwan have PNH clone size more than 50 % (63.6 %), whereas more than ¼ of the PNH patients have clone size smaller than 10 % (27.3 % in Taiwan). The median LDH level at enrollment was 4.9 fold of the upper limit normal (ULN), higher than the ROW (*P* = 0.005). 80 % of the patients had LDH higher than 1.5 fold of ULN in Taiwan, whereas in the ROW around 58.6 % of the patients reached 1.5 fold of ULN (*p* = 0.039). Hemoglobin levels in Taiwan PNH patients were also significantly lower than the ROW (80 g/L vs. 99.0 g/L, *p* < 0.001). Other parameters such as platelets 112 ×10^9^/L, serum creatinine 66.3 umol/L, proteinuria (12.5 % trace, 37.5 % 1+ to 4+, in 36 patients) and eGFR 95.8 mL/min were not significantly different compared to the ROW.

6.7 % (4 out of 60) of the patients in Taiwan had a history of thrombotic events (TE), compared to 13.5 % in the ROW (*p* = 0.178). 12.1 % (7 out of 58) had a history of impaired renal function in Taiwan, similar to 12.6 % observed in the ROW. Symptoms such as shortness of breath (59.3 %), erectile dysfunction (60.7 %) and hemoglobinuria (68.5 %) in Taiwan were more common than the ROW (41.9 %, 23.0 %, and 45.7 % respectively). The symptoms such as fatigue (81.5 %), abdominal pain (31.5 %), and dysphagia (13.0 %) were not different compared to ROW.

### Concomitant medication

Although the hemoglobin levels were lower in Taiwan than the ROW, the history of RBC transfusion in Taiwan PNH patients was 67.8 %, comparable to the ROW (67.8 % vs 57.0 %, *p* = 0.274). The corticosteroid use in Taiwan was significantly more frequent than the ROW (53.2 % vs. 32 %, *p* < 0.001); whereas heparin/warfarin and cyclosporine/ATG use in Taiwan was much lower than the ROW, 3.3 % vs. 19.5 %, 12.9 % vs. 34.2 % respectively (*p* < 0.001) (Table 2).

**Table 2:** Concomitant medication at baseline

	Taiwan Patients (*n* = 63)	ROW (*n* = 3857)	*p*-value
Heparin or Warfarin, *n* (%)	2 (3.3) (*n* = 60)	714 (19.5) (*n* = 3657)	<0.001*
Cyclosporine or ATG, *n* (%)	8 (12.9) (*n* = 62)	1258 (34.2) (*n* = 3682)	<0.001*
Corticosteroids, *n* (%)	33 (53.2) (*n* = 62)	1164 (32.0) (*n* = 3642)	<0.001*

*p <0.05

## Discussion

This is the first time to have a systematic review on the Taiwanese PNH patients. This analysis provides key information of our PNH patients and would definitely help understanding the basic characteristics of this rare disease in Taiwan.

Our analysis of these 63 Taiwan PNH patients in the international PNH registry demonstrates the median LDH level at enrollment was 4.9 fold of the ULN, higher than the ROW 2.0 fold. Lee et al. reported in the similar registry data of 301 Korean PNH patients whose LDH levels were 4.1 fold of the ULN, similar to our finding [5].

In our cohort, the hemoglobin level is 80 g/L, significantly lower than 99 g/L of the ROW. In a large study by Nishimura et al., the 209 Japanese PNH patients also had lower hemoglobin concentration (82 g/L) compared to the Duke patients (97 g/L) [7]. Whether these differences in hemoglobin levels reflect the basic difference between the eastern and western populations needs further investigation in a larger cohort.

In our analysis, some initial symptoms were quite different between ours and the ROW patients. Significantly more PNH patients in Taiwan had classical symptoms of PNH, such as short of breath, hemoglobinuria, erectile dysfunction, while PNH patients in the ROW had higher incidence of thrombosis. The reasons for this discrepancy await for further confirmation in a larger cohort, however the short of breath, hemoglobinuria, and erectile dysfunction could possibly be directly correlated with lower hemoglobin and higher LDH levels in Taiwan.

In a recent study, Lee et al. reported the incidence of thrombosis in the Korean National PNH Registry and demonstrated an incidence of thrombosis of 17.9 % [5]. A retrospective French study of 460 PNH patients also demonstrated 10-year cumulative incidence of 31 % [2]. Our investigation showed a much lower incidence of thrombosis than the previous studies. Recently it has been reported that the incidence of thrombosis in PNH is likely to be underestimated as a result of subclinical pulmonary embolism or myocardial ischemia observed in 6 of 10 patients in one study [8] and evidence of subclinical myocardial damage in 2 of 10 patients in another study [9]. The lower incidence of thrombosis in Taiwan PNH group is consistent with the previous observations. In addition, the lower heparin /warfarin use may reflect less TE observed in Taiwan PNH patients.

The corticosteroid is wildly used in Taiwan PNH patients compared to the patients in the ROW (53.2 % vs 32 %, *P*-value < 0.001). Corticosteroid as a treatment option for both chronic hemolysis and acute hemolytic exacerbations in PNH, is a subject of debate [10–12]. The main value of corticosteroid may be in attenuating acute hemolytic exacerbations. The value of steroid in treating chronic hemolysis is limited by toxicity. Brief pulses of prednisone may reduce the severity and duration of the crisis while avoiding the untoward consequences associated with long term use. However, in the recent study, Jang et al. showed the corticosteroid was the most frequently used medication in their patients (77 %) but there was no difference in mortality between the 104 patients who received corticosteroids for >5.4 years (13 %) and the 197 patients who received corticosteroid for < 5.4 years or had not received corticosteroid at all (15 %; *P* = 0.520) [13]. We should be very cautious about the use of corticosteroid in PNH patients due to the lack of definitive efficacy and the side effects.

## Conclusions

In summary, this is the first systematic review for Taiwan PNH patients. We will need more patients and longer follow-up for better understanding about the characteristics and long-term outcome of PNH patients in Taiwan.

## Abbreviations

CRF: Case report form
PNH: Paroxysmal nocturnal hemoglobinuria
ROW: Rest of the world
TE: Thromboembolism
